# Collagen XI Alpha 1 Chain, a Novel Therapeutic Target for Cancer Treatment

**DOI:** 10.3389/fonc.2022.925165

**Published:** 2022-06-29

**Authors:** Yi-Hui Wu, Cheng-Yang Chou

**Affiliations:** ^1^Department of Medical Research, Chi Mei Medical Center, Tainan, Taiwan; ^2^Department of Nursing, Min-Hwei Junior College of Health Care Management, Tainan, Taiwan; ^3^Department of Obstetrics and Gynecology, National Cheng Kung University Hospital, College of Medicine, National Cheng Kung University, Tainan, Taiwan

**Keywords:** collagen type XI alpha 1, cancer progression, chemoresistance, cancer associated fibroblast activation, epithelial ovarian cancer

## Abstract

The extracellular matrix (ECM) plays an important role in the progression of cancer. Collagen is the most abundant component in ECM, and is involved in the biological formation of cancer. Although type XI collagen is a minor fibrillar collagen, collagen XI alpha 1 chain (COL11A1) expression has been found to be upregulated in a variety of human cancers including colorectal, esophagus, glioma, gastric, head and neck, lung, ovarian, pancreatic, salivary gland, and renal cancers. High levels of COL11A1 usually predict poor prognosis, owing to its association with angiogenesis, invasion, and drug resistance in cancer. However, little is known about the specific mechanism through which COL11A1 regulates tumor progression. Here, we have organized and summarized recent developments regarding the interactions between COL11A1 and intracellular signaling pathways and selected therapeutic agents targeting COL11A1, as these indicate its potential as a target for treatment of cancers, especially epithelial ovarian cancer.

## Introduction

Cancer has become the third leading cause of death worldwide, with approximately 1,918,030 new cancer cases and 609,360 cancer deaths projected to occur in the United States in 2022 (1). Although clinical classification, staging, and treatment guidelines for various cancers are constantly being updated, improvements in the long-term prognosis of cancer patients remain difficult and therefore it is of extreme significance to explore effective cancer treatment strategies. Previously, researchers focused on the biological mechanisms in tumor cells, while ignoring the relationship between cancer cells and the surrounding tissues. Recent studies have focused to a greater extent on the tumor microenvironment, proving that it is crucial in the progression of cancer.

The extracellular matrix (ECM) is a scaffold of fibrillar proteins, accessory proteins, and molecules, and provides structural and biochemical support for cells. The ECM consists of a basement membrane (located at the basal aspect of epithelial or endothelial cells in normal tissues) and the interstitial (stromal) ECM. In most tissues, the basement membrane consists largely of collagen IV, together with laminin, fibronectin, and several types of proteoglycans (2). The main role of the basement membrane is to provide a physical barrier between the epithelial cells and the connective tissue (stroma) of the organ, whilst still allowing the diffusion of gases and transport of signaling molecules. The interstitial ECM, mainly produced by mesenchymal cells, consists primarily of collagens I and III, fibronectin, and proteoglycans. It regulates cell adhesion, cell migration, angiogenesis, and tissue repair (3). In cancer, rupture of the basement membrane permits epithelial cells to undergo epithelial–mesenchymal transition (EMT), migrate into the surrounding stroma, and invade through the interstitial ECM. Epithelial cells that have undergone EMT can cause activation of stromal cells to yield pro-tumorigenic stromal cells, which can in turn remodel the ECM to create a tumor-permissive environment (4). The ECM is indispensable for cell proliferation, differentiation, and maintenance of tissue homeostasis (5), and its influence on cancer cell plasticity is modulated by a variety of cell types that reside within the tumor stroma. Under the influence of systemic regulators as well as cancer cells, these stromal cells produce not only tumor ECM, which qualitatively and quantitatively differs from a normal ECM, but also an array of cytokines and other secreted and membrane-bound factors that influence cancer cell plasticity. Cancer cells, cancer-associated fibroblasts (CAFs), immune cells, adipocytes, and other stromal cells act on the ECM and as a result promote angiogenesis, decrease cell adhesion, induce loss of tissue polarity, and increase EMT. This increases the invasion and distant metastasis of tumor cells and their resistance to chemotherapy drugs; these are generally the leading causes of cancer mortality (6–9).

## Mechanisms Underlying COL11A1 Involvement in Cancer Progression

Collagen type XI alpha 1 (COL11A1) belongs to the collagen family, a major component of the interstitial ECM. Collagen XI is a right-handed triple helix complex consisting of three different α-chains, namely alpha 1, alpha 2, and alpha 3. Although type XI collagen is a minor fibrillar collagen, it is essential for normal tissue integrity and function. The lack of type XI collagen leads to abnormal thickening of the tissue in cartilage (10, 11); animals have been reported to develop chondrodysplasia where the cartilage was devoid of fibrils in two separate mouse models in which collagen XI expression was abrogated (12). These data demonstrate the significant role of collagen XI in nucleation of assembly of short, small-diameter protofibrils. The collagen alpha chain consists of 662–3152 amino acids. *COL11A1* and *COL11A2* encode the alpha 1 chain and alpha 2 chain, respectively; *COL2A1* encodes the alpha 3 chain (10). COL11A1 therefore requires two other alpha chains (COL11A2, COL2A1) to form collagen fibers. COL11A1 expression is absent in benign pathological conditions involving desmoplasia such as hyperplasia, fibrosis, cirrhosis, pancreatitis and inflammatory bowel disease or premalignant lesions (10–13). In contrast, COL11A1 has been found to be upregulated in a variety of cancers including breast, colorectal, esophagus, glioma, gastric, head and neck, lung, ovarian, pancreatic, salivary gland, and renal cancers, and it can be used as a tumor marker to predict the prognosis of cancer (**Table 1**) (14–46). In these cancers, the expression of COL11A1 positively correlates with tumor progression and lymph node metastasis.

**Table 1:** The mechanisms of COL11A1 regulation in different cancers.

Cancer	Regulator	Effect	Reference
Breast	miR-139-5p	Increases cell proliferation and inhibits apoptosis	(14)
	MicroRNA let-7b	Increases cell proliferation, migration, invasion, and metastasis	(15)
	Oncostatin M	Increases inflammation and metastasis	(16)
Colorectal	TGF-β1	Increases cell invasion	(17)
	miR-29	Increases cell invasion	(18)
Esophageal	EMT signal	Increases cell proliferation, migration, invasion	(19)
Gastric	Proliferation genes	Increases cell proliferation, migration, invasion	(20)
Glioma	ERK	Increases cell migration, invasion	(21)
	Histone H3	Increases cell migration, invasion	(22)
Head and neck		Increases cell growth and invasion	(23)
Lung	Smad signal	Increases cell proliferation, migration, invasion, and chemoresistance	(24)
	PI3K/Akt/ERK	Increases cell proliferation, migration and inhibit apoptosis	(25)
	B-Myb	Increases cell proliferation, migration, invasion	(26)
	FGF14	Increases cell proliferation, and migration	(27)
Ovary	TGF-β signal	Increases cell proliferation, migration, invasion, and metastasis	(28–30)
	Akt/c/EBPβ	Increases chemoresistance	(31)
	NF-κB-Twist1	Increases chemoresistance and inhibit apoptosis	(32)
	Akt/NF-κB/IAP	Increases chemoresistance	(33)
	Akt/AMPK-fatty acid oxidation	Increases chemoresistance	(34)
	MFAP5	Increases chemoresistance	(35)
	TGF-β3/NF-κB	Increases cell proliferation, migration and invasion	(36)
	miR-335	Increases cell proliferation, migration, invasion, and chemoresistance	(37)
Pancreas	Mist	Increases cell proliferation, migration, invasion, and chemoresistance	(38)
	GLI1	Poor survival	(39)
	TGF-β signal	Increases cell proliferation, migration, invasion	(40)
	Akt/CREB	Increases chemoresistance and inhibit apoptosis	(41)
Renal	TGF-β signal	Increases cell proliferation and migration	(42)
	miR-200c-5p	Increases cell invasion and inhibit apoptosis	(43)

## Mechanisms Underlying COL11A1 Involvement in CAF Activation

COL11A1 expression has been detected in tumor cells as well as in tumor-associated stromal cells. It has been shown that CAFs, which express and secrete COL11A1 into the ECM, promote the proliferation, angiogenesis, invasion, and drug resistance of cancer cells (10, 17, 28, 47–51). COL11A1 overexpression has only been observed in desmoplastic areas of the tumors, which are composed primarily of CAFs in cancers (28, 29, 48–50). However, such overexpression has not been demonstrated in fibroblasts in inflammatory diseases, making COL11A1 a unique marker for CAFs (10, 48). The malignant transformation of colorectal cancer is associated with the differential expression of COL11A1 in the stroma (49). Navab et al. (52) established CAF primary cultures, matching normal fibroblasts from 15 non–small-cell lung cancer samples, and identified *COL11A1* as the gene with the most highly elevated expression in CAFs. Other studies have also recorded COL11A1 overexpression in CAFs of pancreatic cancer (52) and suggested that it is a CAF-specific marker (48, 53). However, several studies have found that cancer cells secrete the COL11A1 they produce into the ECM, to regulate their own biological behavior as well as that of the surrounding cancer cells (34, 54–57). Recently, we (36) investigated the role of COL11A1 in inducing CAF activation and its interaction with cancer cells. The results showed that human ovarian fibroblasts acquire CAF markers and functions, such as enhanced cell migration and contraction abilities and ECM deposition, and express COL11A1 when cocultured with high COL11A1-expressing epithelial ovarian cancer (EOC) cell lines or conditioned medium of coculture. In contrast, coculturing human ovarian fibroblasts with low COL11A1-expressing EOC cells or *COL11A1* knockdown in EOC cells abrogated the expression levels of COL11A1 and CAF markers in human ovarian fibroblasts. We also demonstrated that COL11A1 regulated transforming growth factor beta 3 (TGF-β3) levels in EOC cells, thereby promoting CAF activation by activating the nuclear factor kappa-light-chain-enhancer of activated B/insulin-like growth factor binding protein-2 (NF-κB/IGFBP2) axis, which was attenuated by COL11A1 knockdown or pharmacological inhibition of TGF-β3. Notably, elevated p-SP1 expression generated by COL11A1-mediated ERK activation induced p65 translocation into the nucleus, thereby enhancing its binding to the *IGFBP2* promoter. TGF-β3 induction by COL11A1 reinforced tumor–fibroblast crosstalk *via* heightened IL-6 production to stimulate the proliferation and invasion of tumor cells. Furthermore, *in vivo* findings from mouse xenografts indicated the essential role of COL11A1 in tumor progression and CAF activation, and that anti-TGF-β3 therapy could inhibit tumor growth and reverse CAF activation. These *in vitro* results were confirmed by clinical findings that high *TGF-β3* mRNA levels are associated with extra-pelvic spread, disease progression, and poor progression-free survival (PFS) and overall survival (OS) of ovarian cancer patients.

As mentioned previously, *COL11A1* encodes one alpha chain out of three alpha chains of type XI collagen; thus, two other alpha chains (COL11A2, COL2A1) are required to form collagen fibers. Interestingly, our results showed that COL2A1 did not activate the CAF phenotype, suggesting that CAF activation may be specific to COL11A1 (36).

## Regulation of COL11A1 in Human Cancer

Multiple studies have proven that COL11A1 participates in cancer progression through various signaling pathways (**Table 1**). COL11A1, as a component of collagen, is mainly present in the extracellular matrix. The mechanism through which it transmits signals through the outside-in pathway is still unclear. Rada et al. (33) demonstrated that integrin α1β1 and discoid domain receptor 2 (DDR2) acted as the receptors for the binding of COL11A1 on cell membranes, activating intracellular signaling pathways, and thus causing cancer progression.

TGF-β1 has been found to regulate COL11A1 in colorectal, pancreatic, and renal cancers, thereby promoting cancer proliferation and invasion (17, 40, 42). We demonstrated that TGF-β1 directly acts on the NF-YA binding site of the promoter of *COL11A1* in ovarian cancer cells, activating COL11A1 and thereby inducing migration and invasion (29). Our results were confirmed by Cheon et al. (30), who demonstrated that the knockdown of COL11A1 reduced the migration and invasion of ovarian cancer cells, which was directly regulated by TGF-β1 signaling. Hida et al. (58) also revealed that NF-YA regulates the promoter region of *COL11A1* in rat chondrosarcoma (RCS) as well as mouse pre-chondrocyte ATDC5 cells.

In addition to TGF-β1, several factors have been shown to regulate COL11A1 expression. Fibroblast growth factor-14 (FGF-14) is downregulated in lung adenocarcinoma patient samples (27), and its overexpression in the lung adenocarcinoma cell line A549 results in downregulation of COL11A1 expression. In addition, the glycoprotein microfibril-associated protein 5 (MFAP5) has been found to enhance COL11A1 expression in ovarian fibroblasts (35). Oncostatin M (OSM), an inflammatory cytokine, has also been shown to bind to collagen type XI after being deposited by neutrophils in MDA-MB-231 breast cancer cell-derived matrices *in vitro* (16). Although this interaction is not known to regulate COL11A1 function, binding, or signaling, OSM has been noted to be immobilized to type XI collagen induced signal transducer and activator of transcription (STAT) signaling in the breast cancer cell line T47D.

Other transcription factors known to regulate COL11A1 expression in the context of cancer include SP1, B-myb, and c/EBPβ. Watanabe et al. (59) showed that Sp1 regulates COL11A1 expression in rodent RCS cells. Jin et al. (26) revealed that B-myb is upregulated in 0 (NSCLC) and B-myb overexpression in H1299 cells leads to COL11A1 overexpression. Matsuo et al. (60) showed that the CCAAT-binding factor CBF/NF-Y, and not c/EBP, positively regulates the transcription of COL11A1 in A204 (a human rhadomyosarcoma cell line) by directly binding to the promoter region of COL11A1. Interestingly, our report (31) demonstrated that anticancer drugs, such as cisplatin and paclitaxel, preferentially induce COL11A1 expression in the cisplatin-resistant ovarian cancer cell line A2780CP70, but not in its cisplatin-sensitive counterpart, the A2780 cell line. Furthermore, knockdown of the transcription factor c/EBPβ attenuated the increase in COL11A1 expression in A2780CP70 cells treated with cisplatin or paclitaxel and phosphoinositide-dependent kinase 1 (PDK1)/Akt signaling activation upregulated c/EBPβ/COL11A1 expression in these cells. These findings suggest that COL11A1 transcription is regulated differentially depending on the cell type.

Some microRNA and long non-coding RNA (lncRNA) are involved in the regulation of COL11A1. miR-139-5p expression is low in breast cancer cells and *COL11A1* is predicted to be a target gene of miR-139-5p. Overexpression of miR-139-5p or silencing of *COL11A1* could inhibit the proliferation of breast cancer cells and promote apoptosis. Simultaneous overexpression of miR-139-5p and *COL11A1* could reverse this effect, indicating that *COL11A1* is a downstream target of miR-139-5p (14). Another study has found that microRNA let-7b is a tumor suppressor, and the binding of CDX2 to let-7b can promote the transcription of let-7b and inhibit the expression of COL11A1, thereby reducing the proliferation, invasion, and migration of breast cancer cells (15). Studies on a nude mouse subcutaneous tumor model have also shown that the overexpression of *CDX2* and let-7b or the knockdown of *COL11A1* could effectively reduce the volume and weight of the tumor (15). Yang et al. (61) demonstrated that the overexpression of microRNA-145 (miR-145) downregulates *COL11A1* expression and that of other chondrocyte markers in the mouse mesenchymal stem cell line C3H10T1/2, while suppression of this microRNA enhances *COL11A1* expression.

There is also evidence that miR-29 downregulates COL11A1 expression during zebrafish development (62). Although no study has directly shown that miR-29 downregulates COL11A1 in mammalian cells, there are at least two studies that predict the role of miR-29 in the downregulation of COL11A1 in a mammalian cell context (63, 64). In addition, protein-protein interaction (PPI) studies have shown that miR-29 affects the progression of colon cancer by targeting COL11A1 (18).

We (37) identified miR-335 as the candidate miRNAs that may regulate COL11A1 expression through an online database search. Further mechanistic experiments revealed that epithelial ovarian cancer (EOC) cell treatment with miR-335 results in phenotypes that mimic those induced by abrogated COL11A1 expression and suppresses cell proliferation and invasion, in addition to increasing the sensitivity of EOC cells to cisplatin. Conversely, treatment with miR-335 inhibitors prompts cell growth/invasiveness and chemoresistance of EOC cells. miR-335 inhibited COL11A1 transcription, thus reducing the invasiveness and chemoresistance of EOC cells *via* the Ets-1/MMP3 and Akt/c/EBPβ/PDK1 axes, respectively. Furthermore, miR-335 did not directly regulate PDK1 but increased PDK1 ubiquitination and degradation through COL11A1 inhibition. *In vivo* findings have shown a significant decrease in the miR-335 expression level in EOC samples compared to that in non-cancerous specimens. Furthermore, patients with low miR335 levels were susceptible to advanced-stage cancer, poor response to chemotherapy, and early relapse. These findings highlighted the importance of miR-335 in downregulating COL11A1-mediated ovarian tumor progression, chemoresistance, and poor survival and suggested the potential application of miR-335 as a therapeutic target (37). Kang et al. (65) demonstrated that COL11A1 promotes esophageal squamous cell carcinoma proliferation and metastasis and is inversely regulated by miR-335-5p.

The lncRNA small nucleolar RNA host gene 12 *(SNHG12)* is overexpressed in a variety of cancers. Xu et al. (43) found that in the renal cancer cells lines A498 and 786O, the apoptosis of cells increased, and the cell survival rate and cell invasion ability decreased when *SNHG12* was knocked out. A luciferase reporter assay indicated that SNHG12 binds to and downregulates miR-200c-5p, thereby increasing COL11A1 expression (43).

## Mechanisms of COL11A1 Participating in Chemoresistance

*COL11A1* is one of the top genes in the gene signature that predicts an outcome to standard chemotherapy in ovarian and other cancers. Our report indicates that ovarian cancer patients who did not respond to standard platinum-based chemotherapies express elevated levels of COL11A1 (31), where COL11A1 was among the top overexpressed genes. Furthermore, high levels of COL11A1 protein secretion have been linked with resistance to platinum-based therapies in ovarian cancer (66). Recently, high levels of circulating COL11A1 have been identified in patients with non-small cell lung cancer (67) and breast cancer (68), which has been correlated with increased aggressiveness of the disease. It is interesting to note that the expression of COL11A1 is elevated post chemotherapy in several cancer types and can mediate resistance to cisplatin chemotherapy (**Table 1**). In lung cancer specimens it has been observed that COL11A1 expression is increased in recurrent tumors (24). In lung cancer cell lines, elevated COL11A1 expression also mediates resistance of cancer cells to cisplatin. Similarly, in ovarian cancer specimens, the expression of COL11A1 is the highest in recurrent tumors compared to primary and metastatic tumors, suggesting that COL11A1 promotes tumor recurrence post chemotherapy (28–30). These studies have been successful in reporting that in human ovarian cancer cell lines and xenograft mouse models COL11A1 is not only associated with poor response to cisplatin, but also confers cisplatin resistance though multiple mechanisms.

We found out that c/EBPβ (CCAAT-enhancer-binding protein-beta) has been identified as a transcription factor upregulating COL11A1 expression post chemotherapy (31). COL11A1 promotes the phosphorylation of SP1, which in turn promotes the transcriptional activation of IKKβ, leading to the increased expression of Twist1 induced by NF-κB, thus causing drug resistance in ovarian cancer cells (32). Another study showed that COL11A1 activates Src- phosphoinositide 3-kinase (PI3K)/Akt-NF-kB signaling to induce the expression of three inhibitor apoptosis proteins (IAPs), including XIAP, BIRC2, and BIRC3. Genetic and pharmacological inhibition of XIAP, BIRC2, and BIRC3 is sufficient to restore cisplatin-induced apoptosis in ovarian cancer cells in the presence of COL11A1 in ovarian cancer cells and xenograft mouse models, respectively (33). Nallanthighal et al. (34) identified a novel role of COL11A1 in modulating tumor cell metabolism. COL11A1 signaling in ovarian cancer cell lines makes them more addicted to fatty acid metabolism to drive cisplatin resistance (34). This ability of COL11A1 to alter the metabolic adaptation of cancer cells benefits the cells to survive the harsh environments generated by chemotherapy.

Overexpression and/or activation of the PI3K/Akt survival pathway have been shown to be associated with carcinogenesis in several tissue types (69–73). One of the main downstream effectors of PI3K is the potent oncogenic serine/threonine kinase Akt, also known as protein kinase B (PKB) (74, 75). Akt is activated by phosphorylation of Thr308 by the PDK1 (76, 77) and phosphorylation of Ser473 by the mammalian target of rapamycin complex 2 (mTORC2) (78). Activated Akt functions to regulate several important molecular pathways, including those associated with cell survival, proliferation, and apoptosis. Our report (31) showed that the level of phosphorylated Akt was elevated in chemoresistant ovarian cancer cells and that treatment with an Akt inhibitor (LY294002) enhanced sensitivity to anticancer drugs. These findings are consistent with previous studies showing that Akt is a determinant of cisplatin resistance in chemoresistant ovarian cancer cells (79–85). Another report from us (31) has revealed that COL11A1 could increase phosphorylated Akt in chemoresistant ovarian cancer cells by stabilizing PDK1 protein. Further evidence of these observations is provided by the reduced PDK1 protein expression observed in COL11A1-knockdown cells, the marked increase in PDK1 protein in the presence of the 26S proteasome inhibitor MG132, and the proteasomal degradation of PDK1 observed in the presence of COL11A1 inhibition. We also observed that phosphorylated Akt levels in chemoresistant ovarian cancer cells were increased by COL11A1 *via* increased binding activity between PDK1 and COL11A1. Collectively, our results demonstrate that the overexpression of COL11A1 leads to chemoresistance, possibly by binding to PDK1-Akt, and subsequently preventing PDK1 degradation. This binding may prevent cisplatin- and paclitaxel-induced PDK1 ubiquitination and degradation in these cells. The results of this study suggest that COL11A1 may be an important determinant of chemoresistance that acts by sequestering PDK1 and preventing its ubiquitination and proteasomal degradation.

Wang et al. (41) demonstrated that COL11A1 phosphorylated AktSer473, promoting proliferation of cancer cells and inhibiting their apoptosis. In pancreatic cancer cells, the COL11A1/Akt axis disrupts the balance between BAX and BCL-2, and inhibits the release of cytochrome C, thereby destroying mitochondrial function and promoting apoptosis escape in turn leading to drug resistance in these cancer cells.

## Targeting COL11A1

As COL11A1 tends to accumulate in tumor tissues and promote malignant progression of human cancer, with lower expression levels in normal tissues, targeting COL11A1 has become an attractive strategy for the treatment of various cancers. Although there is no current therapy specifically designed to target COL11A1 in cancer, there are certain drugs that target COL11A1 signal, some of which have been or are currently being tested in clinical trials (44).

Inhibition of TGF-β, a growth factor known to induce COL11A1 expression, has been explored as an anti-cancer therapy in the clinic. Targeting the TGF-β1 signaling pathway can inhibit metastasis and drug resistance in a variety of human cancer models. LY2157299, a small molecule inhibitor of transforming growth factor beta receptor 1 (TβRI), has entered its phase II clinical trial [86]. LY2157299 inhibits the proliferation and invasion of ovarian cancer cells and inhibits tumor growth in nude mouse xenograft models. In addition, LY2157299 has blocked TGF-β1-induced fibroblast activation and inhibited the expression of COL11A1 in tumor stroma (87). This suggests that LY2157299 may preferably target tumor stroma rather than cancer cells when exerting its anticancer effects. The TβRI kinase inhibitor galunisertib has been evaluated in two phase II clinical trials for hepatocellular carcinoma (HCC), and treatment with galunisertib showed improvement in overall survival in both trials (88, 89). Our present study showed that COL11A1 upregulates *IGFBP2* transcription by augmenting p65 DNA-binding activity in ovarian cancer cells to constitutively activate the TGF-β3 signaling pathway, thereby promoting CAF activation. These findings signify the intriguing possibility that anti-TGF-β1 therapeutics could be used to target ovarian cancer cells and anti-TGF-β3 therapies could be used to inhibit CAFs in COL11A1-positive tumors (36).

*Solanum incanum* extract (also known as SR-T100) has been shown to downregulate c/EBPβ and COL11A1 expression, thereby sensitizing melanoma and ovarian cancer cells to cisplatin (90, 91). In addition to targeting COL11A1 transcription, it might also be possible to target COL11A1 post-translation. HSP47 has been shown to be a molecular chaperone required for procollagen folding in the ER (92). Ito et al. (93) screened small-molecule compounds that inhibit Hsp47’s interaction and chaperone activity with collagen and found that a compound AK778 and its cleavage product Col003 disrupted HSP47/collagen binding and also inhibited collagen secretion.

Akt signaling pathway is closely related to cancer progression, metastasis, and drug resistance (94). COL11A1 is involved in the activation of Akt, which in turn promotes the transcription of COL11A1 (95, 96). Therefore, inhibitors of Akt may be candidates for targeting COL11A1. Our report showed that SC66, an Akt inhibitor, was found to inhibit the proliferation of various human ovarian cancer cells *in vitro*, and the sensitivity of ovarian cancer cells to SC66 was negatively correlated with the content of COL11A1 in cells. SC66 can also increase the sensitivity of ovarian cancer cells to cisplatin and paclitaxel. After SC66 treatment, the invasive ability of ovarian cancer cells was found to have decreased significantly. SC66 has been shown to inhibit the binding of NF-YA and c/ERBβ to COL11A1 promoter, thereby reducing the chemotherapy resistance induced by Twist1 and Mcl-1, and inhibiting the expression of *MMP3* and the invasion ability of cancer cells. However, MK-2206 did not regulate the promoter activity changes of COL11A1 (95). After testing three compounds synthesized from AstraZeneca (Cambridge, UK), it was found that only AZD5653 could inhibit the mRNA content and promoter activity of COL11A1, while AZD8835 and AZD8186 inhibited the phosphorylation of Akt, but had no effect on COL11A1. These results indicated that AZD5663 and not the other two inhibitors inhibit COL11A1 transcription by blocking PDK1 activity (96). In conclusion, the selection of appropriate Akt inhibitors is essential for the inhibition of COL11A1.

Targeting inhibitor of apoptosis (IAPs) proteins, which have been shown to be upregulated by COL11A1 (33), might be another way to target the COL11A1 signaling. Two phase I clinical trials have demonstrated that IAP inhibitors ASTX660 and APG-1387 were well tolerated by patients (97). Another IAP inhibitor LCL161 (a Smac mimetic) was also well tolerated in a phase II clinical trial for myelofibrosis, a form of leukemia (98). Another clinical trial using birinapant, an XIAP/cIAP1 antagonist, to treat high-grade serous ovarian cancer showed that birinapant was well tolerated and downregulated tumor cIAP1 (99).

## Conclusions

In this review, we provide a comprehensive overview of the biological functions of COL11A1 in cancer and discuss how COL11A1 mediates the crosstalk between cancer cells and the tumor microenvironment (TME) to regulate the phenotypes of cancer cell and CAF. Despite our increased understanding of COL11A1 functions in CAF, it remains unclear whether COL11A1 secreted by cancer cells and CAFs has structural and functional similarities. Further evidence is needed to show how COL11A1 regulates the biophysical properties of tumor ECM and how it affects the invasion, migration, and the proliferation of tumor and immune cells. COL11A1 overexpression has been shown to upregulate chemoresistance, and the roles of COL11A1 in cancer stemness, tumor dormancy, inflammation, and recurrence remain unclear.

**Figure 1:**
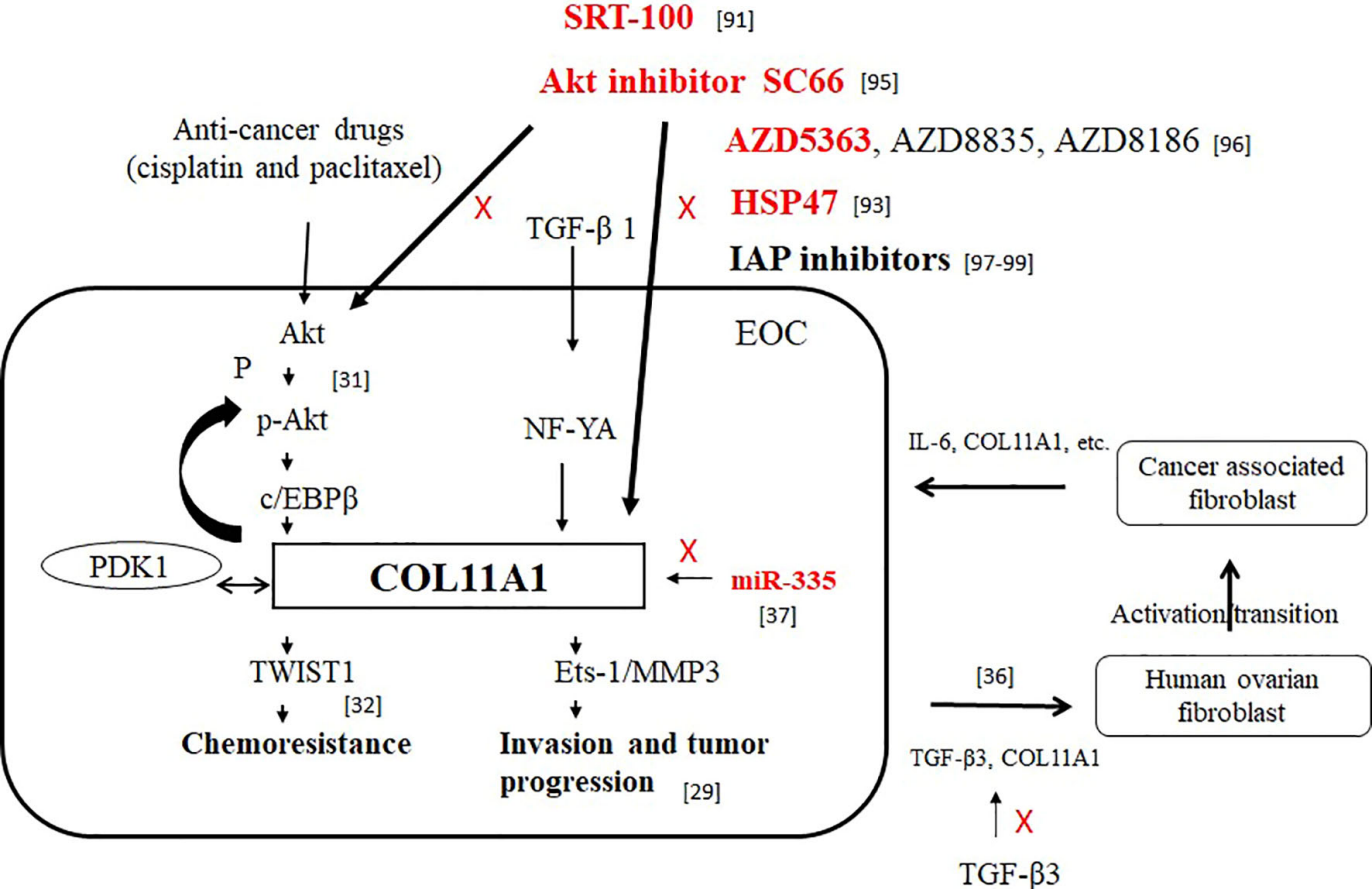
The roles of COL11A1 and Akt as predictive markers in the prognosis of cancer and as drug design targets in EOC. Red font indicates inhibition of COL11A1 expression.

The PI3K/Akt signaling pathway has been investigated as a critical regulator of cancer cell survival, and a number of Akt pathway inhibitors with different efficacies and specificities have been identified. We speculate that Akt inhibitors may exert their effect on Akt signaling through different mechanisms and the evaluation of PI3K/Akt/mTOR pathway inhibitors is required to confirm the patterns of sensitivity observed in preclinical studies before they can be applied to clinical settings. In our opinion, COL11A1 and Akt play roles as predictive markers in the prognosis of cancer and as drug design targets in epithelial ovarian cancer (**Figure 1**).

## Author Contributions

Y-HW: original draft preparation; C-YC: supervision. All authors have read and agreed to the published version of the manuscript.

## Funding

This work was supported by grants from the National Science Council (MOST: No. 108-2314-B-384-011-MY3, 108-2314-B-006-061-MY2 and 110-2314-B-006-036). The study was also supported by grants from the Chi Mei Medical Center, Liouying Campus (CLFHR10822, CLFHR10911, CMLMOST10901, CLFHR10921, CLFHR11015 and CMLMOST11101).

## Conflict of Interest

The authors declare that the research was conducted in the absence of any commercial or financial relationships that could be construed as a potential conflict of interest.

## Publisher’s Note

All claims expressed in this article are solely those of the authors and do not necessarily represent those of their affiliated organizations, or those of the publisher, the editors and the reviewers. Any product that may be evaluated in this article, or claim that may be made by its manufacturer, is not guaranteed or endorsed by the publisher.

## Abbreviations

COL11A1, collagen type XI alpha 1; CAF, cancer associated fibroblast activation; EOC, epithelial ovarian cancer; ECM, extracellular matrix; EMT, epithelial–mesenchymal transition; TGF-β3, transforming growth factor beta 3; NF-κB/IGFBP2, nuclear factor kappa-light-chain-enhancer of activated B/insulin-like growth factor binding protein-2; PFS, progression-free survival; OS, overall survival; DDR2, discoid domain receptor 2; MFAP5, microfibril-associated protein 5; OSM, Oncostatin M; STAT, signal transducer and activator of transcription; NSCLC, non-small cell lung cancer; PDK1, phosphoinositide-dependent kinase 1; lncRNA, long non-coding RNA; PPI, protein-protein interaction; SNHG12, small nucleolar RNA host gene 12; c/EBPβ, CCAAT-enhancer-binding protein-beta; PI3K, phosphoinositide 3-kinase; IAP2, inhibitor apoptosis protein; PKB, protein kinase B; TβRI, transforming growth factor beta receptor 1; HCC, hepatocellular carcinoma.
